# Cerebral phosphoester signals measured by ^31^P magnetic resonance spectroscopy at 3 and 7 Tesla

**DOI:** 10.1371/journal.pone.0248632

**Published:** 2021-03-18

**Authors:** Shizhe Li, Jan Willem van der Veen, Li An, JoEllyn Stolinski, Christopher Johnson, Maria Ferraris-Araneta, Milalynn Victorino, Jyoti Singh Tomar, Jun Shen

**Affiliations:** 1 National Institute of Mental Health, Bethesda, MD, United States of America; 2 National Institute of Neurological Disorders and Stroke, Bethesda, MD, United States of America; CIC bioGUNE, SPAIN

## Abstract

Abnormal cell membrane metabolism is associated with many neuropsychiatric disorders. Free phosphomonoesters and phosphodiesters, which can be detected by in vivo ^31^P magnetic resonance spectroscopy (MRS), are important cell membrane building blocks. However, the quantification of phosphoesters has been highly controversial even in healthy individuals due to overlapping signals from macromolecule membrane phospholipids (MP). In this study, high signal-to-noise ratio (SNR) cerebral ^31^P MRS spectra were acquired from healthy volunteers at both 3 and 7 Tesla. Our results indicated that, with minimal spectral interference from MP, the [phosphocreatine (PCr)]/[phosphocholine (PC) + glycerophosphocholine (GPC)] ratio measured at 7 Tesla agreed with its value expected from biochemical constraints. In contrast, the 3 Tesla [PCr]/[PC+GPC] ratio obtained using standard spectral fitting procedures was markedly smaller than the 7 Tesla ratio and than the expected value. The analysis suggests that the commonly used spectral model for MP may fail to capture its complex spectral features at 3 Tesla, and that additional prior knowledge is necessary to reliably quantify the phosphoester signals at low magnetic field strengths when spectral overlapping is significant.

## Introduction

^31^P magnetic resonance spectroscopy (MRS) allows the evaluation of several important aspects of brain energetics and metabolism, from levels of ^31^P-containing metabolites to metabolic fluxes associated with adenosine triphosphate (ATP)-generating enzymes. Since the 1970s, many studies have also used ^31^P MRS and functional ^31^P MRS techniques to investigate a broad range of both normal brain functions and neuropsychiatric disorders [1–5]. These ^31^P MRS studies have provided considerable insight into the role of mitochondrial metabolism and cell membrane synthesis in normal physiology and disease development as well as therapeutic interventions.

Phospholipids are known to have a phosphodiester (PDE) structure whose major resonances overlap with the resonances of free PDE and phosphomonoester (PME). The in vivo phospholipid signals are very pronounced at low magnetic fields [6, 7]. These signals, often referred to as membrane phospholipids (MP), originate from ^31^P-containing macromolecules, including phospholipid bilayers. They decrease with increasing magnetic field strength [7–9]. At 7 Tesla and higher magnetic field strengths, the signals of glycerophosphocholine (GPC), glycerophosphoethanolamine (GPE), phosphocholine (PC), and phosphoethanolamine (PE) are well resolved, with little or no spectral interference from the much reduced MP signals [8, 9]. However, at the more commonly used 3 Tesla or lower field strengths, large MP signals have consistently been observed [10, 11]. Typically, spectral fitting techniques are necessary to extract the individual components of PDE, PME, and inorganic phosphates (P_i_). With proton decoupling, the MP signals at 1.5 Tesla were found to be broad and highly asymmetrical [10]. High resolution ^31^P nuclear magnetic resonance (NMR) studies of lipid extracts of animal brain also revealed multiple ^31^P signals resonating at different frequencies [12]. Nevertheless, it has been customary to use a single symmetrical peak centered at 2.3 ppm to fit the in vivo MP resonances. This is of concern because many pathological conditions involving abnormal cell membrane metabolism may alter the composition of the MP signals and, by inference, their lineshape [13].

The detrimental effects of the strong macromolecule signals on accurate quantification of short echo time proton MRS spectra using the LCModel have recently been recognized [14]. Because the separation between small metabolite molecules and macromolecules by spectral fitting relies on differences in their linewidths, the broad linewidths of the composite PDE and PME signals at 1.5–3 Tesla may make it difficult to reliably remove spectral contamination from MP. As an example, the reported PDE/ATP ratio in brain ranged from 0.4 to 4.0, as measured in healthy adult volunteers using 1.5–3 Tesla [10, 15–20]; a span of this order of magnitude cannot be reconciled by potential differences in MRS techniques, T_1_ saturation, nuclear Overhauser enhancement, or variations in spatial distribution of phosphoesters.

In this work we acquired high signal-to-noise ratio (SNR) cerebral ^31^P MRS spectra from healthy subjects at both 3 and 7 Tesla. We found that the [PCr]/[PC+GPC] ratio extracted from 7 Tesla ^31^P MRS spectra of healthy subjects is consistent with biochemical constraints from the literature. In comparison, the 3 Tesla [PCr]/[PC+GPC] ratio obtained using standard spectral fitting procedure is significantly smaller than the expected value. Our results suggest that the commonly used spectral model of MP may not be suitable for quantifying the overlapping phosphoester signals at low magnetic field strengths.

## Materials and methods

The human study was performed under the protocol NCT01266577 that was approved by the National Institutes of Health (NIH) Institutional Review Board (IRB). Written consent forms were obtained from all participants.

### 7 Tesla ^31^P MRS

**Fig 1:**
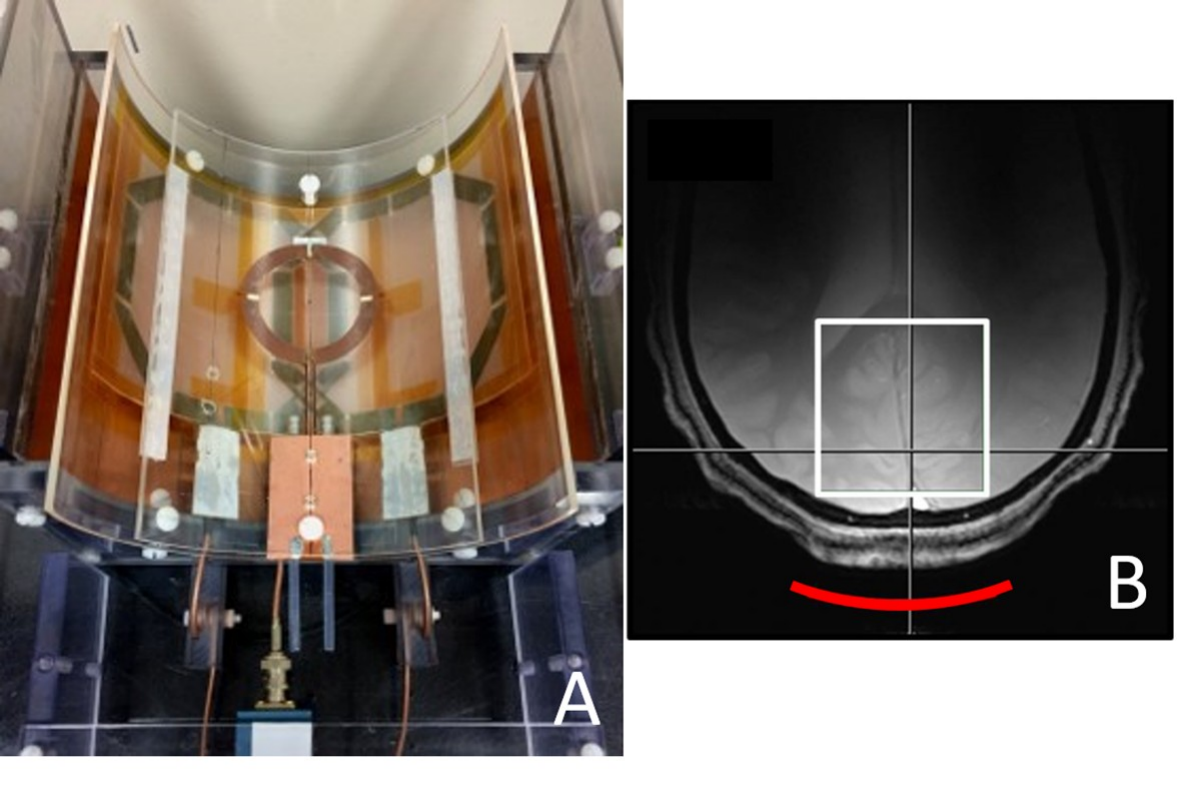
A 7 Tesla radio frequency (RF) coil assembly and its relative position to the human head. (A) The circular ^31^P surface coil, the ^1^H coil (two overlapping octagonal loops), and the RF shield were mounted on the bottom surface of the upper, middle, and lower tubes, respectively. Each proton loop had a ^1^H cable trap constructed using an RG-316 cable. A ^31^P cable trap, built inside an RF shielded box, was connected to the ^31^P coil. (B) Gradient echo localizer image from a healthy volunteer showing the relative position of the ^31^P surface coil (red arc, 7.5 cm dia.) and the shim voxel (5 x 5 x 5 cm^3^ box in while color).

In vivo ^31^P MRS experiments were performed on a Siemens Magnetom 7 Tesla scanner (Siemens Healthcare, Erlangen, Germany). A ^1^H and ^31^P coil assembly was built in-house (Fig 1A), comprising a circular ^31^P coil (diameter = 7.0 cm), a quadrature half-volume proton coil, and a slotted radio frequency (RF) shield. These RF devices were mounted on three vertically stacked, thin, semi-cylindrical plastic tubes. A ^31^P surface coil was mounted on the outer surface of the upper tube (outer diameter = 20.3 cm). A ^1^H frequency blocking L-C tank circuit was placed at the middle point of the ^31^P circular loop. The proton coil, which consisted of two overlapped octagon loops with nominal length/width of 12.3 cm, was mounted on the outer surface of the middle tube (outer diameter = 20.3 cm), and the slotted RF shield was mounted on the inner surface of the lower tube (inner diameter = 22.2 cm). Each proton loop had a single-tuned ^1^H cable trap constructed using an RG-316 cable. A ^1^H/^31^P dual-tuned cable trap, built inside an RF-shielded box, was connected to the ^31^P coil. The coil assembly was connected to the 7 Tesla scanner via an interface box (Quality ElectroDynamics, Mayfield Village, Ohio, USA) that included transmit-receive switches, pre-amplifiers, RF filters for both channels, and a quadrature combiner for the proton channel.

^31^P MRS data were acquired from six healthy volunteers (mean age: 30 years; age range: 22–48 years). A voxel of 5 x 5 x 5 cm^3^ located right above the ^31^P coil (Fig 1B) was selected for performing B_0_ shimming in the occipital lobe using the Siemens 3D shim tool with correction of all first- and second-order shims and five third-order shims. Shimming results were evaluated by measuring the water linewidth (full width at half height) from the 5 x 5 x 5 cm^3^ voxel using proton MRS with a point resolved spectroscopy (PRESS) sequence. Water linewidth was found to be 13.9 ± 1.2 Hz (n = 6). ^31^P spectra were acquired using the Siemens excite-acquire sequence: hard pulse = 250 μs, number of data points = 1024, and spectral width (SW) = 5 kHz. The transmit frequency was set to the frequency of PCr at 0 ppm. Two repetition time (TR) were used to evaluate the effect of T_1_ saturation. At TR = 3 seconds, the number of averages (NA) was 64, and at TR = 30 seconds, the NA was 32 (n = 6 for both TRs). The transmit voltage for ^31^P was pre-optimized in vivo to obtain maximum signal-to-noise ratio for a given TR.

### 3 Tesla ^31^P MRS

In vivo ^31^P MRS scans were also performed on a Siemens Skyra 3 Tesla scanner (Siemens Healthcare, Erlangen, Germany). A 3 Tesla ^1^H and ^31^P coil assembly was built in-house that had the same geometrical layout as the 7 Tesla coil assembly described above except that the coils were tuned to 3 Tesla frequencies and there was no RF shield. The coil assembly was connected to the 3 Tesla scanner via an interface box (Stark Contrast MRI Coils Research, Erlangen, Germany). Eight heathy volunteers (mean age = 29 years, age range: 21–45 years) were recruited and scanned. The same shimming procedure used in the 7 Tesla MRS was applied to optimize field homogeneity of a 4 x 4 x 4 cm^3^ voxel prescribed above the ^31^P surface coil without the third order corrections. Water linewidth was found to be 15.5 ± 0.9 Hz (n = 8). ^31^P spectra were acquired using the Siemens excite-acquire sequence: hard pulse = 500 μs, number of data points = 1024, and SW = 5 kHz. The transmit frequency was set to the frequency of PCr at 0 ppm. At TR = 2 seconds, the NA was 128, and at TR = 25 seconds, the NA was 64 (n = 8 for both TRs). The ^31^P RF power at 3 Tesla was calibrated in the same way as in the 7 Tesla experiments.

### ^31^P MRS spectral fitting

Raw data were first preprocessed using an IDL (Harris Geospatial Solutions, Boulder, CO, USA) program to zero the first two (TR = 2, 3 seconds) or three (TR = 25, 30 seconds) complex data points in free induction decay for baseline suppression. The chemical shift of phosphocreatine (PCr) was set to 0 ppm. The data were subsequently phase-corrected and saved in the Siemens RDA raw data format. The RDA data were then processed using the jMRUI software package with the Amares routine [21] and prior spectral knowledge of ^31^P metabolites [21–24]. The ^31^P basis set consisted of α-, β-, γ-adenosine triphosphate (α-, β-, γ-ATP), nicotinamide adenine dinucleotide (NAD), PCr, MP, GPC, GPE, intracellular inorganic phosphate (P_i_), extracellular inorganic phosphate (P_i_ extra), PC, PE, and uridine diphosphate glucose (UDPG). Four different spectral models of MP (Lorentzian, Gaussian lineshapes, fixed and freely adjustable linewidths) were evaluated to assess the effects of MP on ^31^P MRS data quantification. For MP fitting with fixed linewidths, the fixed linewidth values were calculated from the median of the freely fitted MP linewidths.

### [PCr]/[GPC+PC]

The choline singlet at 3.2 ppm observed in the proton MRS spectrum of healthy volunteers originates almost entirely from the trimethylamine group of PC and GPC [10, 25], given that the concentrations of choline, acetylcholine, and phosphatidylcholine in their free forms are negligibly low at the sensitivity level of proton MRS [26–28]. That is,

[tCho]=[GPC+PC] (1)

where tCho is total choline. The ratio of total creatine (tCr) to total choline ([tCr]/[tCho]) and [PCr]/[GPC+PC] can be obtained from proton and ^31^P MRS data, respectively, without absolute quantification. Because of Eq 1,

([PCr]/[GPC+PC])/([tCr]/[tCho])=[PCr]/[tCr] (2)

The creatine kinase equilibrium is tightly regulated, with [PCr] ≈ [Cr] predicted by the creatine kinase equilibrium constant and in vitro assays [10, 29]. The methylene proton signals of PCr and creatine (Cr) resonate at 3.93 and 3.91 ppm, respectively, in the proton MRS spectra. MRS studies of rodent brain performed at very high magnetic field strengths (≥ 9.4 Tesla) provided visual confirmation that the spectrally resolved PCr and Cr methylene protons were of similar intensity. Previous studies noted that the [PCr]/[tCr] ratio in the cerebral cortex was 0.46–0.59 from the spectrally resolved PCr and Cr in proton MRS spectra acquired at 9.4–16.4 Tesla [30–32], with an average value of 0.53. From the above analysis, the expected [PCr]/[GPC+PC] ratio is given by

[PCr]/[GPC+PC]≈0.53*[tCr]/[tCho] (3)

Because the reported phosphoester levels of healthy volunteers in the ^31^P MRS literature vary over an order of magnitude (0.4–4.0) [10, 15–20], the relationship given by Eq 3 may be used as an approximate internal standard to check ^31^P spectral fitting of data acquired from healthy volunteers.

## Results

### 7 Tesla ^31^P MRS

**Fig 2:**
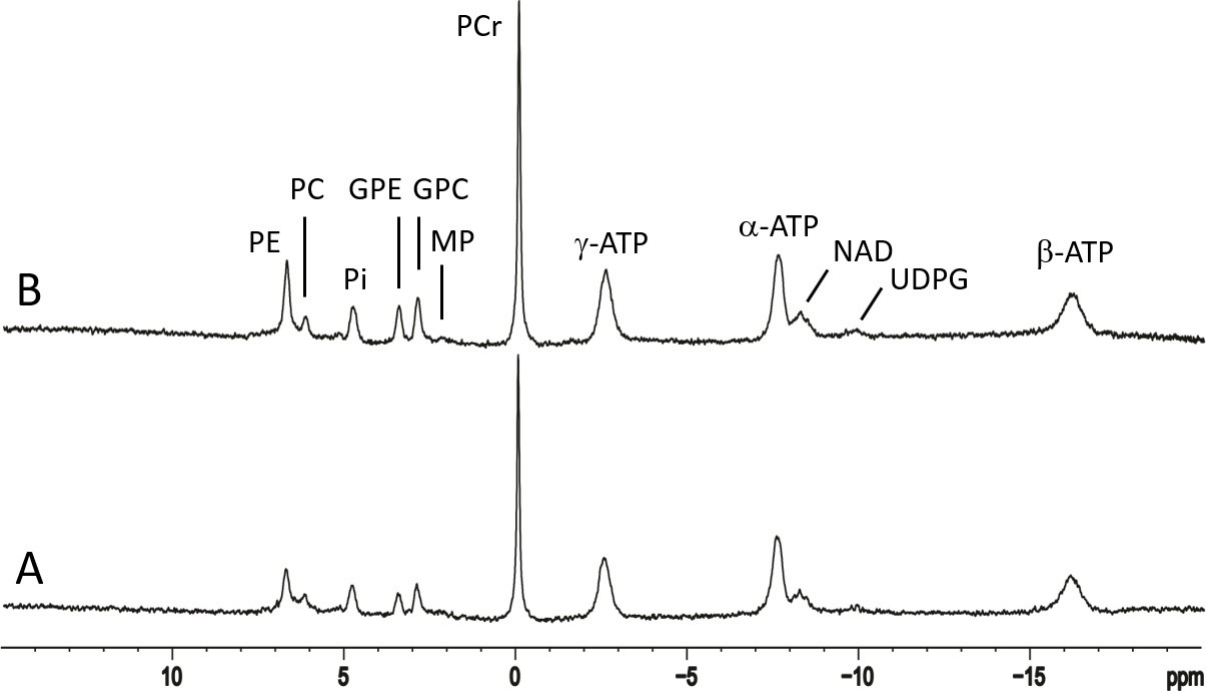
Typical 7 Tesla ^31^P spectra of a healthy volunteer. A 7 Tesla spectrum at (A) TR = 3 seconds, NA = 64 and (B) TR = 30 s, NA = 32 x 2. PE: phosphoethanolamine; PC: phosphocholine; Pi: inorganic phosphate; GPE: glycerophosphoethanolamine; GPC: glycerophosphocholine; MP: membrane phospholipids; PCr: phosphocreatine; α-, β-, γ-ATP, α-, β-, γ-adenosine triphosphate; NAD: nicotinamide adenine dinucleotide; UDPG: uridine diphosphate glucose.

**Fig 3:**
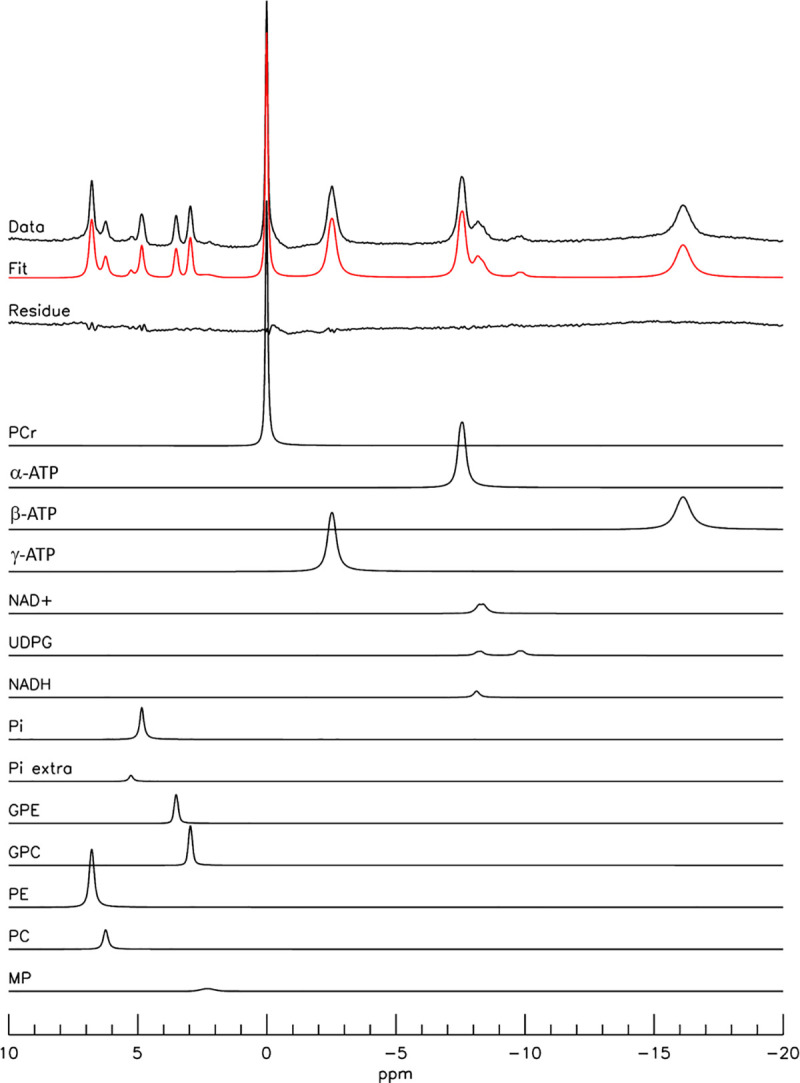
jMRUI analysis of a typical ^31^P spectrum acquired at 7 Tesla. The original spectrum was from Fig 2B with TR = 30 seconds, NA = 64. The individual spectral components, the overall fit and fit residual were also shown. A Gaussian curve with freely adjustable linewidth was used to fit the MP signal at 2.3 ppm. PCr: phosphocreatine; α-, β-, γ-ATP: α-, β-, γ-adenosine triphosphate; NAD^+^: oxidized nicotinamide adenine dinucleotide; UDPG: uridine diphosphate glucose; NADH: reduced form of nicotinamide adenine dinucleotide; Pi: inorganic phosphate; Pi extra: extracellular inorganic phosphate; GPE: glycerophosphosethanolamine; GPC: glycerophosphocholine; PE: phosphoethanolamine; PC: phosphocholine; MP: membrane phospholipids.

Typical 7 Tesla in vivo ^31^P spectra from the occipital lobe of human brain are shown in Fig 2, where spectrum (A) was acquired with TR = 3 seconds and NA = 64, and (B) with TR = 30 seconds and NA = 64. Resonances of PE (6.8 ppm), PC (6.2 ppm), P_i_ (4.8 ppm), GPE (3.5 ppm), GPC (2.9 ppm), MP (2.3 ppm), PCr (0 ppm), γ-ATP (-2.5 ppm), α-ATP (-7.6 ppm), NAD, UDPG P_β_ (-9.8 ppm), and β-ATP (-16.2 ppm) were detected. The signal of oxidized NAD (NAD^+^, -8.2 and -8.4 ppm) and reduced form of NAD (NADH, -8.1 ppm) were collectively labeled as NAD. Notably, the MP signals at 2.3 ppm were quite weak and spectrally resolved from GPC at 2.9 ppm. The intensity variations between the two TR settings were expected from the known T_1_ values of ^31^P-containing metabolites [33]. An example of jMRUI fitting of a 7 Tesla ^31^P spectrum acquired with TR = 30 seconds is shown in Fig 3, where the MP signals at 2.3 ppm were modeled using a Gaussian curve with freely adjustable linewidth. Table 1 lists the corresponding concentrations of ^31^P-containing metabolites (normalized to [γ-ATP]) extracted from jMRUI fitting for both TR = 3 seconds and 30 seconds (see S1–S4 Figs in Supplementary Materials for additional examples of jMRUI fitting of 7 Tesla spectra and a comparison of spectral analysis results using the four different spectral models of MP described in Materials and Methods). From the jMRUI analysis results given in Table 1, [PCr]/[PC+GPC] was found to be 2.4 ± 0.3 (TR = 3 seconds, n = 6) and 2.6 ± 0.3 (TR = 30 seconds, n = 6). The [MP]/[γ-ATP] ratio was found to be 0.13 ± 0.09 (TR = 3 seconds, n = 6) and 0.03 ± 0.04 (TR = 30 seconds, n = 6), consistent with the small MP signals observed at 2.3 ppm in Fig 2.

**Table 1:** ^31^P metabolite concentration ratios determined at 7 Tesla (n = 6).

^31^P Metabolite Ratios	TR = 3 seconds	TR = 30 seconds
	Mean, SD	Mean, SD
[PE]/[γ-ATP]	0.51, 0.04	0.59, 0.02
[PC]/[γ-ATP]	0.22, 0.02	0.19, 0.03
[P_i_]/[γ-ATP]	0.40, 0.03	0.33, 0.03
[GPE]/[γ-ATP]	0.16, 0.01	0.19, 0.02
[GPC]/[γ-ATP]	0.25, 0.01	0.28, 0.03
[MP]/[γ-ATP]	0.13, 0.09	0.03, 0.04
[PCr]/[γ-ATP]	1.14, 0.11	1.22, 0.12
[NAD]/[γ-ATP]	0.18, 0.04	0.19, 0.05
[PCr]/[PC+GPC]	2.42, 0.27	2.60, 0.29

A Gaussian spectral model with freely adjustable linewidth was used to model MP.

TR: repetition time; PE: phosphoethanolamine; PC: phosphocholine; Pi: inorganic phosphate (intracellular inorganic phosphate plus extracellular inorganic phosphate); GPE: glycerophosphoethanolamine; GPC: glycerophosphocholine; MP: membrane phospholipids; PCr: phosphocreatine; γ-ATP: γ-adenosine triphosphate; NAD: nicotinamide adenine dinucleotide.

### 3 Tesla ^31^P MRS

**Fig 4:**
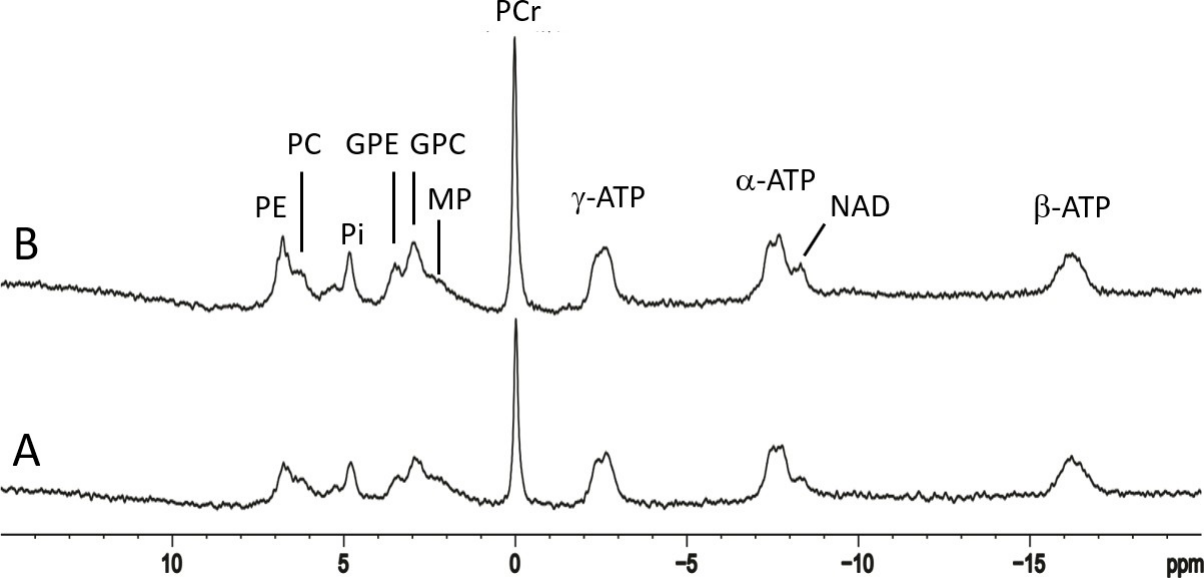
Typical 3 Tesla ^31^P spectra of a healthy volunteer. A 3 Tesla spectrum at (A) TR = 2.5 seconds, NA = 128 and (B) TR = 25 seconds, NA = 128. PE: phosphoethanolamine; PC: phosphocholine; Pi: inorganic phosphate; GPE: glycerophosphoethanolamine; GPC: glycerophosphocholine; MP: membrane phospholipids; PCr: phosphocreatine; α-, β-, γ-ATP: α-, β-, γ-adenosine triphosphate; NAD: nicotinamide adenine dinucleotide.

**Fig 5:**
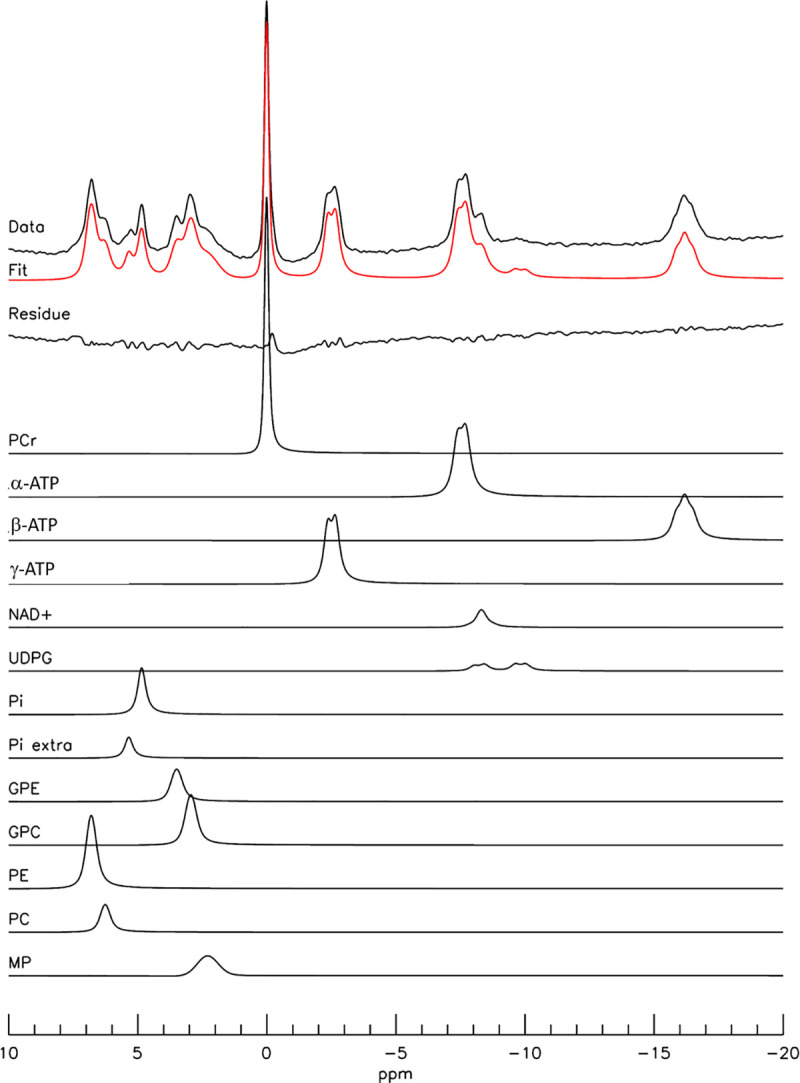
jMRUI analysis of a typical ^31^P spectrum acquired at 3 Tesla. The original spectrum was from Fig 4B with TR = 25 seconds, NA = 64. The individual spectral components, the overall fit and fit residual were also shown. A Gaussian curve with freely adjustable linewidth was used to fit the MP signal at 2.3 ppm. PCr: phosphocreatine; α-, β-, γ-ATP: α-, β-, γ—adenosine triphosphate; NAD^+^: oxidized nicotinamide adenine dinucleotide; UDPG: uridine diphosphate glucose; Pi: inorganic phosphate; Pi extra: extracellular inorganic phosphate; GPE: glycerophosphosethanolamine; GPC: glycerophosphocholine; PE: phosphoethanolamine; PC: phosphocholine; MP: membrane phospholipids.

Typical 3 Tesla ^31^P spectra acquired from a healthy volunteer are shown in Fig 4 at TR = 2 seconds (A) and 25 seconds (B); each spectrum was averaged with NA = 128. Resonances of PE, PC, P_i_, GPE, GPC, PCr, NAD, and α-, β-, and γ-ATP were detected although the individual components of PME and PDE were not spectrally resolved. In contrast to the nearly flat baseline at 7 Tesla seen in Figs 2 and 3, a prominent baseline was observed in the 1–7 ppm region in the 3 Tesla ^31^P MRS spectra (Fig 4). From TR = 2 seconds to TR = 25 seconds, the relative increase in signal intensities of ^31^P metabolites measured at 3 Tesla was similar to the 7 Tesla results. An example of jMRUI fitting of a 3 Tesla ^31^P spectrum acquired with TR = 25 seconds, where the MP signal at 2.3 ppm was also modeled using a Gaussian curve, is shown in Fig 5. The corresponding concentrations of ^31^P-containing metabolites (normalized to [γ-ATP]) extracted from jMRUI fitting of the 3 Tesla spectra are listed in Table 2 for both TR = 2 seconds and 25 seconds. The [PCr]/[PC+GPC] ratio extracted from our 3 Tesla data was 1.1 ± 0.1 (TR = 2 seconds, n = 8) and 1.3 ± 0.3 (TR = 25 seconds, n = 8). The fitted [MP]/[γ-ATP] ratio was found to be 0.77 ± 0.27 (TR = 2 seconds, n = 8) and 0.43 ± 0.22 (TR = 25 seconds, n = 8) at 3 Tesla, which is substantially greater than the values detected at 7 Tesla. Notably, the 3 Tesla [PCr]/[PC+GPC] ratio extracted from spectral analysis using LCModel [24] was similar to our jMRUI results obtained at the same field strength. For additional examples of 3 Tesla spectral fitting and a comparison of spectral analysis results using the four different spectral models of MP at 3 Tesla see S5–S8 Figs in Supplementary Materials.

**Table 2:** ^31^P metabolite concentration ratios determined at 3 Tesla (n = 8).

^31^P Metabolite Ratios	TR = 2 seconds	TR = 25 seconds
	Mean, SD	Mean, SD
[PE]/[γ-ATP]	0.78, 0.06	0.86, 0.09
[PC]/[γ-ATP]	0.37, 0.04	0.31, 0.06
[P_i_]/[γ-ATP]	0.82, 0.14	0.66, 0.12
[GPE]/[γ-ATP]	0.36, 0.03	0.38, 0.07
[GPC]/[γ-ATP]	0.56, 0.05	0.63, 0.09
[MP]/[γ-ATP]	0.77, 0.27	0.43, 0.22
[PCr]/[γ-ATP]	1.09, 0.17	1.22, 0.12
[NAD]/[γ-ATP]	0.15, 0.07	0.23, 0.05
[PCr]/[PC+GPC]	1.16, 0.16	1.33, 0.26

A Gaussian spectral model with freely adjustable linewidth was used to model MP.

TR: repetition time; PE: phosphoethanolamine; PC: phosphocholine; Pi: inorganic phosphate (intracellular inorganic phosphate plus extracellular inorganic phosphate); GPE: glycerophosphoethanolamine; GPC: glycerophosphocholine; MP: membrane phospholipids; PCr: phosphocreatine; γ-ATP: γ-adenosine triphosphate; NAD: nicotinamide adenine dinucleotide.

**Fig 6:**
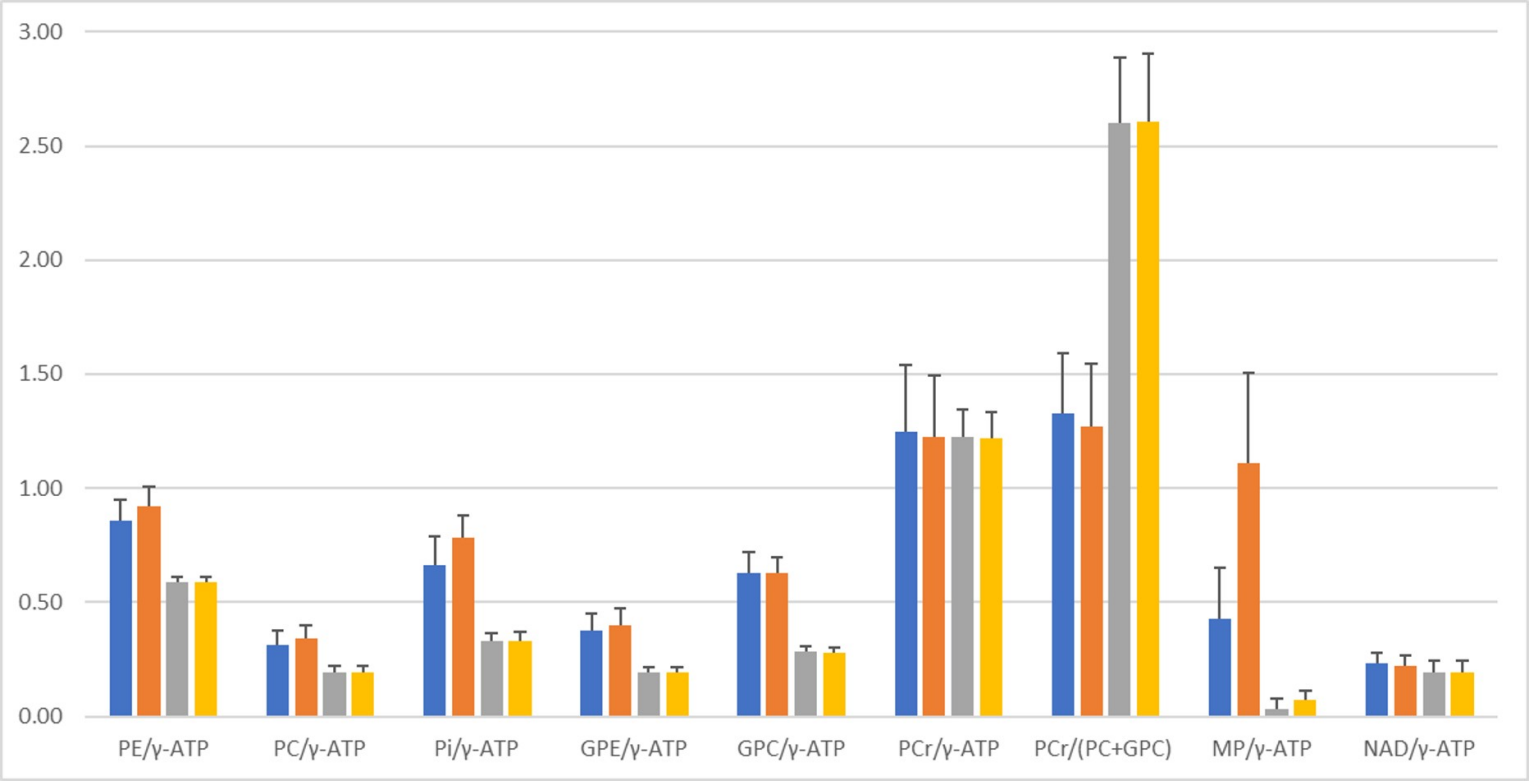
Comparison of [^31^P metabolite]/[γ-ATP] ratios measured at 3 Tesla and 7 Tesla. 3 Tesla spectra (TR = 25 seconds) and 7 Tesla spectra (TR = 30 seconds) were fitted with freely adjustable linewidths for MP. Blue: 3 Tesla, fitted using Gaussian lineshape; orange: 3 Tesla, fitted using Lorentzian lineshape; grey: 7 Tesla, fitted using Gaussian lineshape; yellow: 7 Tesla, fitted using Lorentzian lineshape.

Fig 6 compares [^31^P metabolite]/[γ-ATP] ratios measured at 3 and 7 Tesla with long TRs and freely adjustable linewidths for MP. Student *t*-tests were performed for the values averaged over the four spectral models of MP (see Table 3). Although there was a general agreement among the upfield signals (PCr, α-, β-, γ-ATP, and NAD) across field strengths and MP lineshape models, significant differences were seen between 3 and 7 Tesla downfield [^31^P metabolite]/[γ-ATP] ratios. The differences between the fitted 3 and 7 Tesla downfield [^31^P metabolite]/[γ-ATP] ratios remain highly significant after Bonferroni corrections of the p values are made.

**Table 3:** Student *t*-test results for 3 Tesla (n = 8) and 7 Tesla (n = 6) ^31^P metabolite ratios.

^31^P Metabolite Ratios*	p-value^¶^
[PE]/[γ-ATP]	6.2 E-06
[PC]/[γ-ATP]	7.4 E-05
[P_i_]/[γ-ATP]	7.7 E-08
[GPE]/[γ-ATP]	2.5 E-05
[GPC]/[γ-ATP]	1.3 E-06
[MP]/[γ-ATP]	6.6 E-05
[PCr]/[γ-ATP]	7.8 E-01
[NAD]/[γ-ATP]	1.9 E-01
[PCr]/[PC+GPC]	4.1 E-06

*Calculated by averaging fitting results obtained from the four different spectral models of MP.

^**¶**^Student *t*-tests (two-tailed, unpaired) without Bonferroni corrections. PE: phosphoethanolamine; PC: phosphocholine; Pi: inorganic phosphate (intracellular inorganic phosphate plus extracellular inorganic phosphate); GPE: glycerophosphoethanolamine; GPC: glycerophosphocholine; MP: membrane phospholipids; PCr: phosphocreatine; γ-ATP: γ-adenosine triphosphate; NAD: nicotinamide adenine dinucleotide.

### Comparison with Eq 3

The total creatine to total choline ratio [tCr]/[tCho] has been well documented in a large number of in vivo proton MRS and chemical shift imaging studies, and results from various studies broadly agree [34, 35]. Because the volume of brain tissue measured by ^31^P MRS is usually large, we averaged tCr and tCho values from the existing literature [34, 35] and arrived at [tCr]/[tCho] ≈ 4.8 for cerebral cortex of healthy adult volunteers (see Table 4). The [PCr]/[GPC+PC] ratio expected from Eq 3 is therefore approximately 0.53*4.8 = 2.5.

**Table 4:** Literature values used to calculate the expected cerebral [PCr]/[GPC+PC] ratio using Eq 2.

Metabolite concentration ratios	References
[PCr]/[tCr] = 0.59	[31]
[PCr]/[tCr] = 0.53	[32]*
[PCr]/[tCr] = 0.46	[30]
[tCr]/[tCho] = 7.85/1.70 = 4.62	[34]^†^
[tCr]/[tCho] = (8.39+6.39)/(1.44+1.70) = 4.71	[35]^§^
[tCr]/[tCho] = (8.36+6.27)/(1.35+1.58) = 4.99	[35]^¶^

*Measured from hippocampus

^†^Medians of the reported concentrations

^§^Unweighted average as described in ref. [35]. The values from pure gray and pure white matter were averaged

^¶^Weighted average as described in ref. [35]. The values from pure gray and pure white matter were averaged.

In short echo proton MRS, spectral fitting of the macromolecule baseline using LCModel was found to be strongly influenced by SNR and linewidth [14]. Despite the large number of ^31^P MRS studies reported in the literature, our comparison was limited to studies of healthy volunteers that measured PCr, GPC, and PC intensities using a large voxel or localization by surface coil for maximum SNR [10, 33, 36, 37]. General agreement was observed between the experimental [PCr]/[PC+GPC] ratios measured at 7 Tesla and that expected from Eq 3 (Table 5). Thus, it appears that one of the key advantages of 7 Tesla is that PE, PC, GPE, GPC, and MP can be spectrally resolved due to the dramatically reduced MP intensity and increased chemical shift dispersion at the much higher magnetic field strength.

**Table 5:** Comparison of [PCr]/[PC+GPC] ratios.

[PCr]/[PC+GPC]	References
2.5	Current Study (Eq 3)
2.5	10 (1.5 Tesla, ^1^H-decoupled, MP removed, averaged over young and elderly adults)
2.3	36 (1.5 Tesla, ^1^H-decoupled, MP removed)
2.3	37 (1.5 Tesla, ^1^H-decoupled, MP removed)
2.7	33 (7 Tesla)
2.6 ± 0.3	Current study (7 Tesla, TR = 30 s, n = 6)
2.4 ± 0.3	Current study (7 Tesla, TR = 3 s, n = 6)
1.3	24 (3 Tesla, LCModel and jMRUI)*
1.3 ± 0.3	Current study (3 Tesla, TR = 25 s, n = 8)
1.2 ± 0.2	Current study (3 Tesla, TR = 2 s, n = 8)

GPC: glycerophosphocholine; PC: phosphocholine; PCr: phosphocreatine.

*[PCr]/[PC+GPC] ratio was estimated from Fig 4 in ref. [24].

For lower magnetic field strengths, proton decoupling is needed to improve spectral resolution of ^31^P MRS to resolve the phosphoesters acquired from large tissue volumes [10]. Due to safety concerns and nonstandard hardware requirements, few previous studies attempted proton decoupling. In particular, the 1.5 Tesla studies listed in Table 5 employed proton decoupling, nuclear Overhauser enhancement, and MP baseline removal with PE, PC, GPE, and GPC peaks fully resolved. The [PCr]/[PC+GPC] ratios obtained from those 1.5 Tesla studies agreed reasonably well with our 7 Tesla and Eq 3 results.

Although spectral fitting can extract individual components of MRS signals, sufficient prior knowledge is crucial when significant spectral overlapping is present. Because the ground truth lineshape of MP at 3 Tesla is unknown, a single symmetrical MP basis peak centered at 2.3 ppm is unlikely to fully capture the complex MP signals. The 3 Tesla [PCr]/[PC+GPC] ratios extracted using both LCModel [24] and jMRUI ([24], and Tables 2 and 5 of the current study) were ~50% of the value expected from Eq 3; thus, both are markedly smaller than the [PCr]/[PC+GPC] ratios determined from studies with spectrally resolved phosphoesters (Tables 1 and 5).

## Discussion

This study, which found that the [PCr]/[PC + GPC] ratio measured at 7 Tesla, but not 3 Tesla, agreed with values expected from biochemical constraints. The results suggest that a single symmetrical peak may fail to capture the complex spectral features of MP at 3 Tesla, and that additional prior knowledge is necessary to reliably quantify the downfield ^31^P signals at low magnetic field strengths when spectral overlapping is significant.

The first two or three data points of the FIDs were discarded in this study, a practice commonly used in processing ^31^P MRS data in order to suppress the broad baseline (e.g., [33]). Since the spectral width of our study is 5 kHz, this is equivalent to adding a time delay of 400–600 μs before recording the FIDs. This procedure has no significant effect on quantification of MP as the time delay corresponds to signals with kHz linewidths.

Abnormal levels of PME and PDE—both of which are cell membrane building blocks—have been associated with many neuropsychiatric disorders [38, 39]. In particular, the PDE/PME ratio was found to be elevated in cancer patients and to fall with effective treatment [5]; the PDE/PME ratio has also been considered a marker of MP turnover equilibrium, which is altered in many diseases, including cancer [10, 38]. Furthermore, cell line and tumor model studies have found that PC/GPC and PE/GPE ratios, which appear to be significantly higher in primitive neuroectodermal tumors [4], may serve as indicators of malignancy.

Despite the importance of these markers, ^31^P MRS studies in many neuropsychiatric disorders that involve phosphoesters have obtained mixed results. For example, many ^31^P MRS studies reported disturbances in phospholipid metabolism in Alzheimer’s disease that correlated with pathological biomarkers [40]; in contrast, other studies [1] found no changes in metabolite concentrations or ratios in patients compared with controls in whole axial sections of the brain and no correlation between ^31^P MRS indexes and severity of dementia. Notably, it is likely that contamination by MP has contributed to many of the mixed findings.

In the present study, the low [PCr]/[PC+GPC] ratio measured at 3 Tesla is consistent with significant spectral contamination by MP that inflates its denominator while PCr is affected very little due to its narrow linewidth and to the large resonant frequency separation between PCr and MP. Therefore, when the [PCr]/[PC+GPC] ratio extracted from the ^31^P MRS spectra of healthy volunteers is conspicuously smaller than the value expected from Eq 3, MP contamination should be suspected. Caution is needed if the same spectral fitting procedure is to be applied to data acquired from patients since their MP lineshape may have been altered under pathological conditions. It also should be noted that Eq 3 is derived for healthy subjects. When data from healthy controls are unavailable additional experiments including complementary ^1^H MRS studies of patients are needed to validate spectral fitting procedures at 3 Tesla or lower magnetic field strengths.

The advent of 7 Tesla clinical scanners provides an opportunity to revisit many of the early controversies because not only is MP signal intensity greatly reduced at 7 Tesla, it is also spectrally resolved from GPE and GPC. Furthermore, PE, PC, GPC, and GPE are all resolved at 7 Tesla with reasonable B_0_ shimming without the need to perform proton decoupling. Despite these advantages, most ^31^P clinical studies are expected to continue to be conducted on the more prevalent 3 Tesla scanners and without proton decoupling for the foreseeable future. Importantly, our analysis of spectral fitting with symmetrical MP models suggests that additional prior knowledge is required to avoid significant contamination of downfield signals, as evidenced by the lower [PCr]/[PC+GPC] ratios obtained from both LCModel [24] and jMRUI (see Tables 2 and 5) analyses of 3 Tesla ^31^P data.

In addition to potential contamination by MP, other factors may also affect metabolite quantification by ^31^P MRS. For example, nuclear Overhauser enhancement is not uniform across the ^31^P signals [41]; specifically, in contrast to the nearly uniform T_1_ of most metabolites detected by proton MRS [42], there is a large dispersion in T_1_s of ^31^P MRS signals [33, 43], making their intensities sensitive to the commonly used TR values. Such variations may also have contributed to the different quantification results reported in the ^31^P MRS literature. Furthermore, although it is relatively easy to use the same MRS parameter settings for control and patient groups to reduce variability, MP contamination is more worrisome as many diseases may also alter MP signals, as may specific changes in cell membrane metabolism [13]. Therefore, it is important to minimize or eliminate spectral contamination from MP signals when free phosphoesters are of clinical interest.

Many metabolite ratios reported in Tables 1 and 2 may not be generalized because the current study used surface transceiver coils to acquire ^31^P MRS spectra from a small number of human subjects and no gradient-based spatial localization techniques were employed. Because of the severe B_1_ inhomogeneity associated with surface coils the ^31^P signals are expected to be affected by many factors specific to our experiments such as head size, size of the surface coil, RF calibration, and placement of transmitter frequency. For example, many ratios reported in Table 1 are significantly different from the corresponding values reported by a similar 7 Tesla study that used a larger surface coil [33]. Nevertheless, it should be noted that the [PCr]/[PC+GPC] ratio is insensitive to spatial location of ^31^P signals for the same tissue type due to the intrinsic biological constraints contained in Eq [3]. As such it is expected that this internal standard is relatively immune to many variations originated from the use of surface transceiver coils.

Taken together, the results of the present study suggest that contamination by MP signals is minimal at 7 Tesla because of the diminished intensity of MP and increased spectral resolution at high magnetic fields. In contrast, the free phosphoester signals in ^31^P MRS spectra acquired at low magnetic fields (≤ 3 Tesla) overlap with the large MP signals. Although MP lineshape has been revealed to be highly asymmetrical at 1.5 Tesla with proton decoupling [35], the true lineshape and spectral pattern of the complex MP signal at 3 Tesla remain undetermined. The biochemical constraints described here suggest that fitting the MP signals at 3 Tesla using a symmetrical peak centered at 2.3 ppm leads both to its underestimation and to a significant overestimation of phosphoesters. In contrast, the [PCr]/[PC+GPC] ratio expected from Eq 3 agrees reasonably well with 1.5 Tesla data acquired with MP removal and 7 Tesla data, lending confidence to characterization of membrane metabolism in brain disorders using 7 Tesla ^31^P MRS without proton decoupling.

## Conclusion

When spectral interference from MP is minimized, the experimentally measured [PCr]/[PC+GPC] ratios were found to be in reasonable agreement with the value expected from biochemical constraints. The common practice of fitting the complex MP signal by a single symmetrical peak can lead to overestimation of phosphoester signals at low magnetic field strengths including 3 Tesla.

## Supporting information

S1 Fig ^31^P spectral fitting of the 7 Tesla spectrum shown in Fig 2B with TR = 30 seconds. A Gaussian curve with fixed linewidth of 160 Hz was used to fit the MP signal.
(TIF)

S2 Fig ^31^P spectral fitting of the 7 Tesla spectrum shown in Fig 2B with TR = 30 seconds. A Lorentzian curve with a freely adjustable linewidth was used to fit the MP signal.
(TIF)

S3 Fig ^31^P spectral fitting of the 7 Tesla spectrum shown in Fig 2B with TR = 30 seconds. A Lorentzian curve with a fixed linewidth of 320 Hz was used to fit the MP signal.
(TIF)

S4 Fig ^31^P metabolite ratios extracted from 7 Tesla spectra at TR = 30 seconds. The MP signals were fitted using the four spectral models: Blue: Gaussian with a fixed linewidth of 160 Hz; orange: Gaussian with a freely adjustable linewidth; grey: Lorentzian with a fixed linewidth of 320 Hz; yellow: Lorentzian with a freely adjustable linewidth.
(TIF)

S5 Fig ^31^P spectral fitting of the 3 Tesla spectrum shown in Fig 4B with TR = 25 seconds. A Gaussian curve with a fixed linewidth of 100 Hz was used to fit the MP signals.
(TIF)

S6 Fig ^31^P spectral fitting of the 3 Tesla spectrum shown in Fig 4B with TR = 25 seconds. A Lorentzian curve with a freely adjustable linewidth was used to fit the MP signals.
(TIF)

S7 Fig ^31^P spectral fitting of the 3 Tesla spectrum shown in Fig 4B with TR = 25 seconds. A Lorentzian curve with a fixed linewidth of 200 Hz was used to fit the MP signals.
(TIF)

S8 Fig ^31^P metabolite ratios extracted from 3 Tesla spectra TR = 25 seconds. The MP signals were fitted using the four spectral models: Blue: Gaussian with a fixed linewidth of 100 Hz; orange: Gaussian with a freely adjustable linewidth; grey: Lorentzian with a fixed linewidth of 200 Hz; yellow: Lorentzian with a freely adjustable linewidth.
(TIF)
