# Supplementary figures and images for: Cerebral phosphoester signals measured by 31P magnetic resonance spectroscopy at 3 and 7 Tesla
Source: PLoS One. 2021 Mar 18;16(3):e0248632. doi: 10.1371/journal.pone.0248632 (PMC7971532; doi:10.1371/journal.pone.0248632)

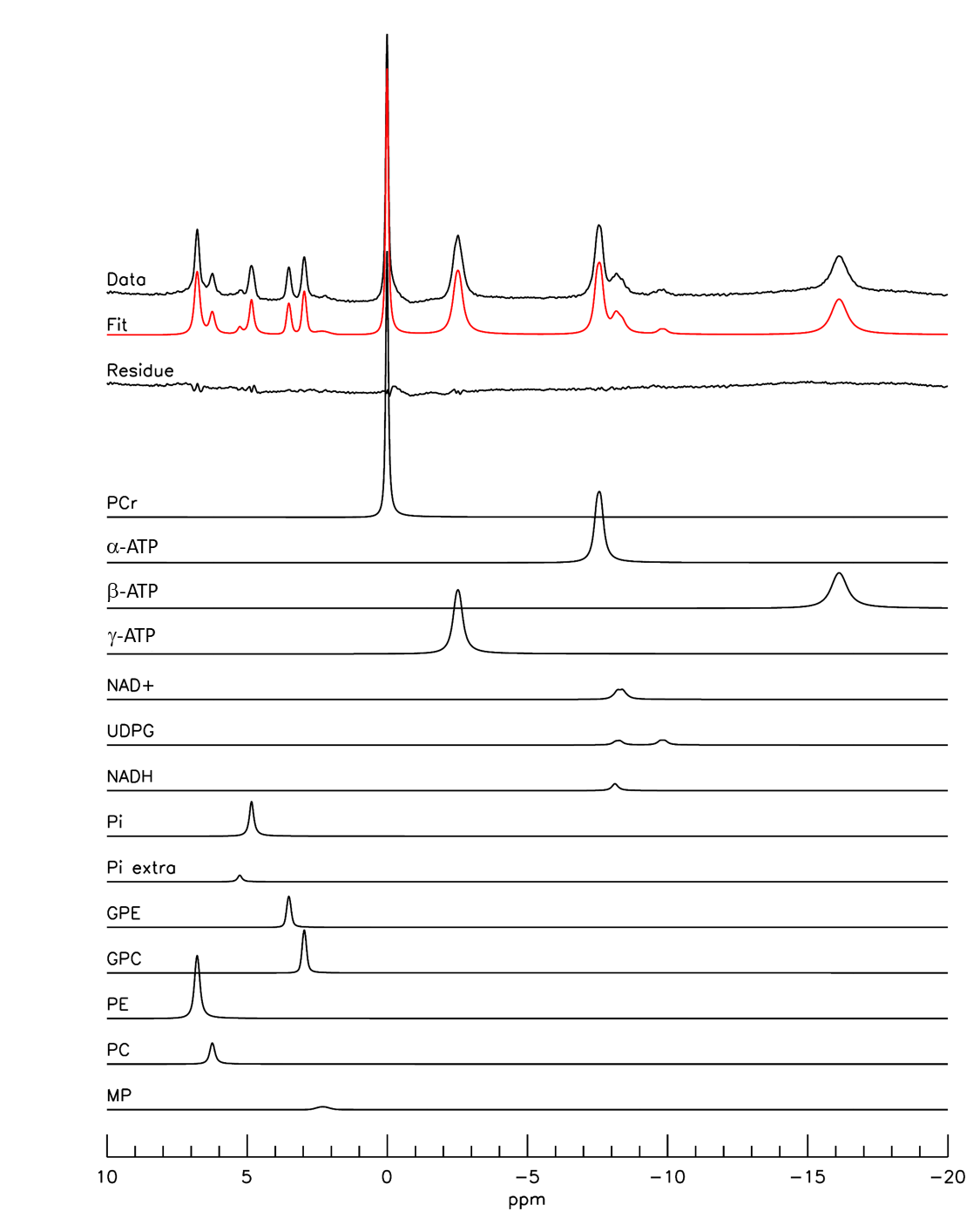

Supplement: S1 Fig — A Gaussian curve with fixed linewidth of 160 Hz was used to fit the MP signal. (TIF) [file pone.0248632.s001.tif]

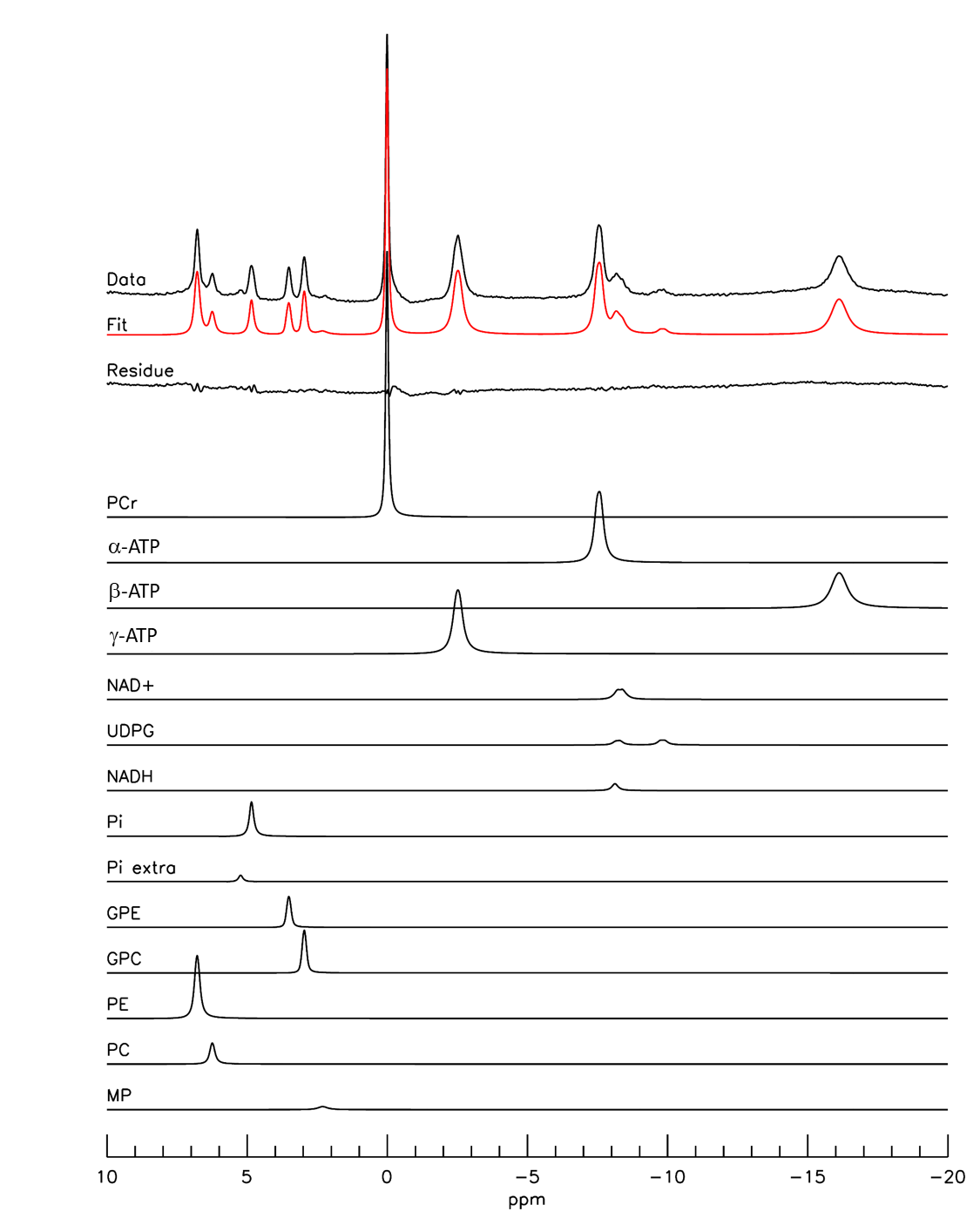

Supplement: S2 Fig — A Lorentzian curve with a freely adjustable linewidth was used to fit the MP signal. (TIF) [file pone.0248632.s002.tif]

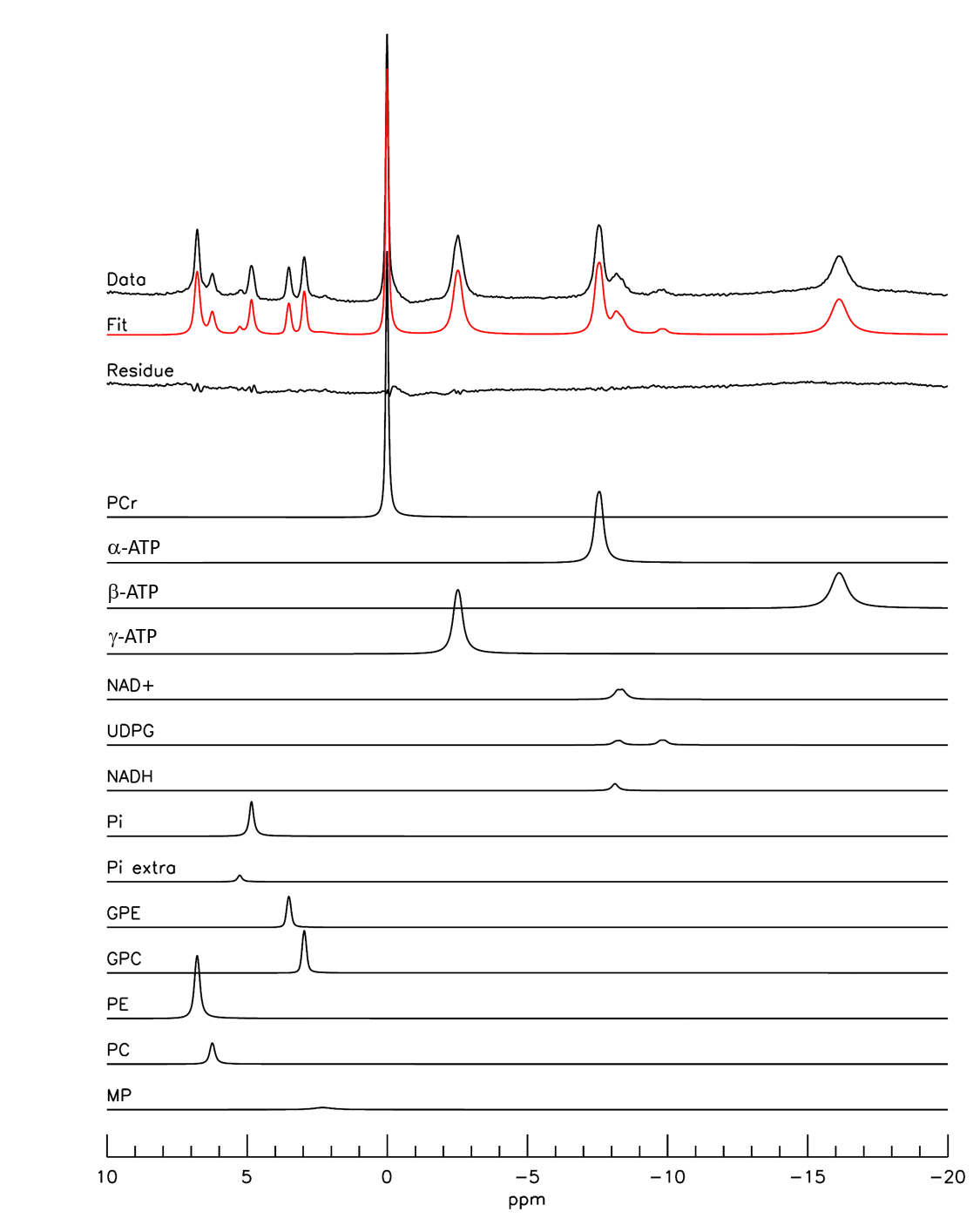

Supplement: S3 Fig — A Lorentzian curve with a fixed linewidth of 320 Hz was used to fit the MP signal. (TIF) [file pone.0248632.s003.tif]

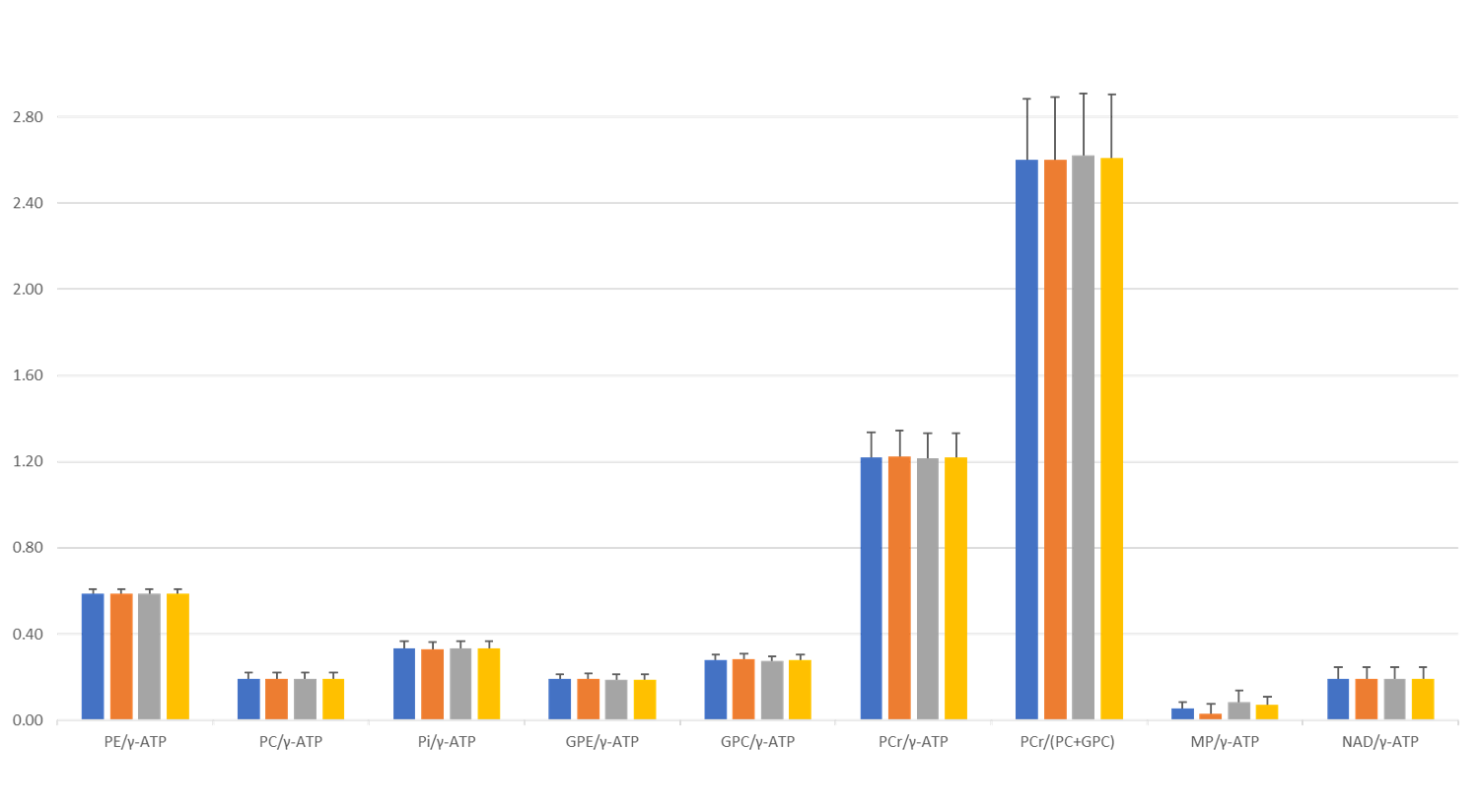

Supplement: S4 Fig — The MP signals were fitted using the four spectral models: Blue: Gaussian with a fixed linewidth of 160 Hz; orange: Gaussian with a freely adjustable linewidth; grey: Lorentzian with a fixed linewidth of 320 Hz; yellow: Lorentzian with a freely adjustable linewidth. (TIF) [file pone.0248632.s004.tif]

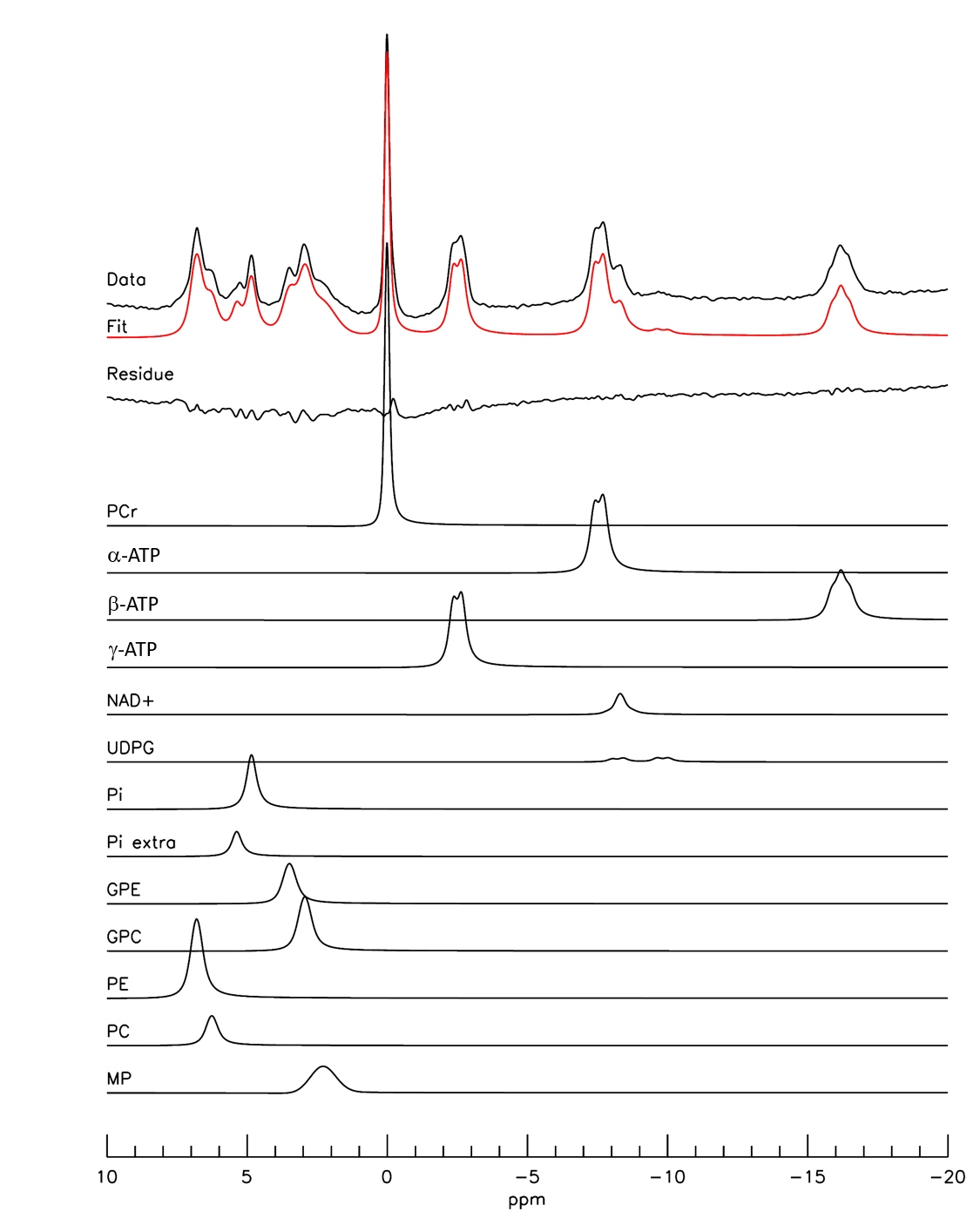

Supplement: S5 Fig — A Gaussian curve with a fixed linewidth of 100 Hz was used to fit the MP signals. (TIF) [file pone.0248632.s005.tif]

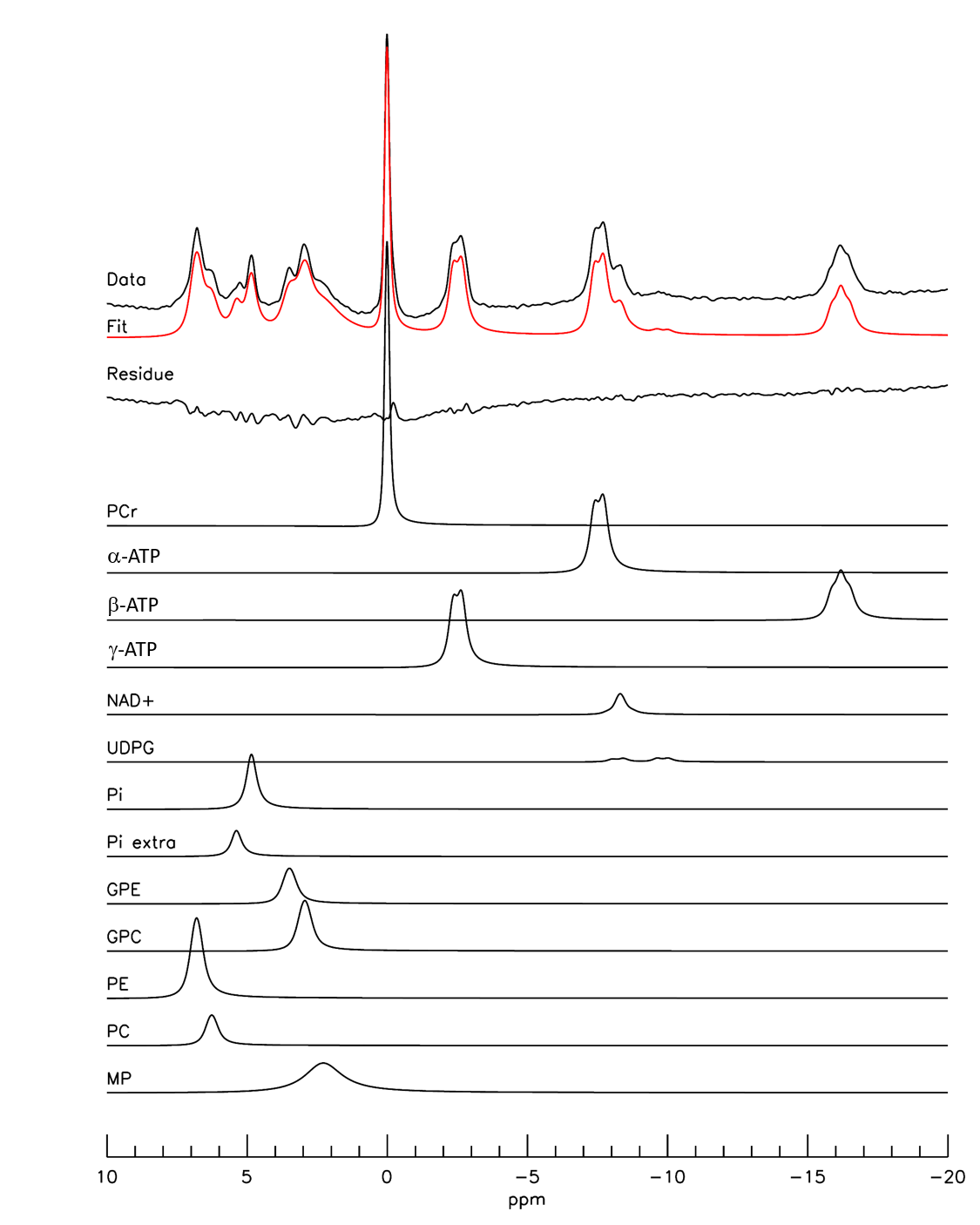

Supplement: S6 Fig — A Lorentzian curve with a freely adjustable linewidth was used to fit the MP signals. (TIF) [file pone.0248632.s006.tif]

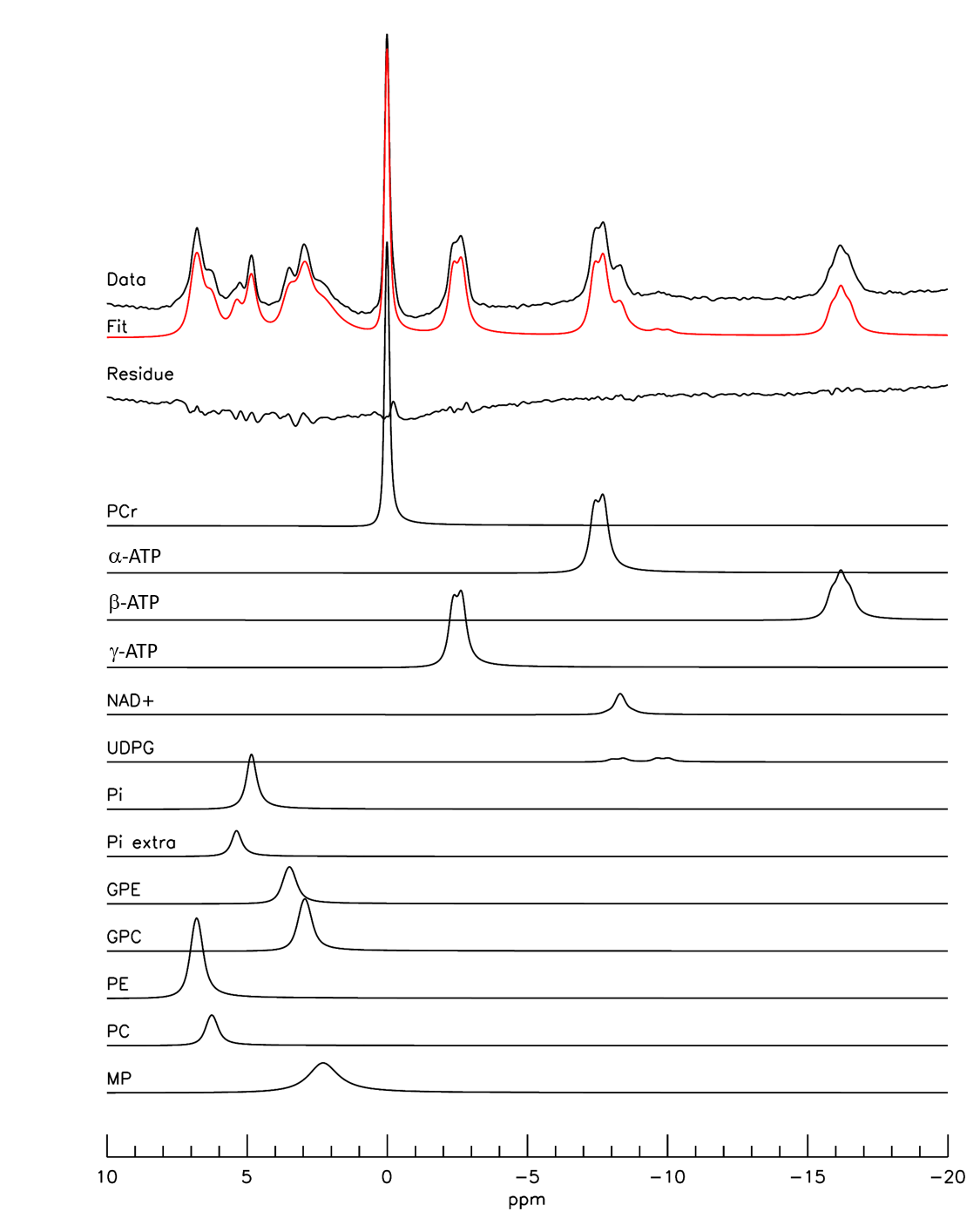

Supplement: S7 Fig — A Lorentzian curve with a fixed linewidth of 200 Hz was used to fit the MP signals. (TIF) [file pone.0248632.s007.tif]

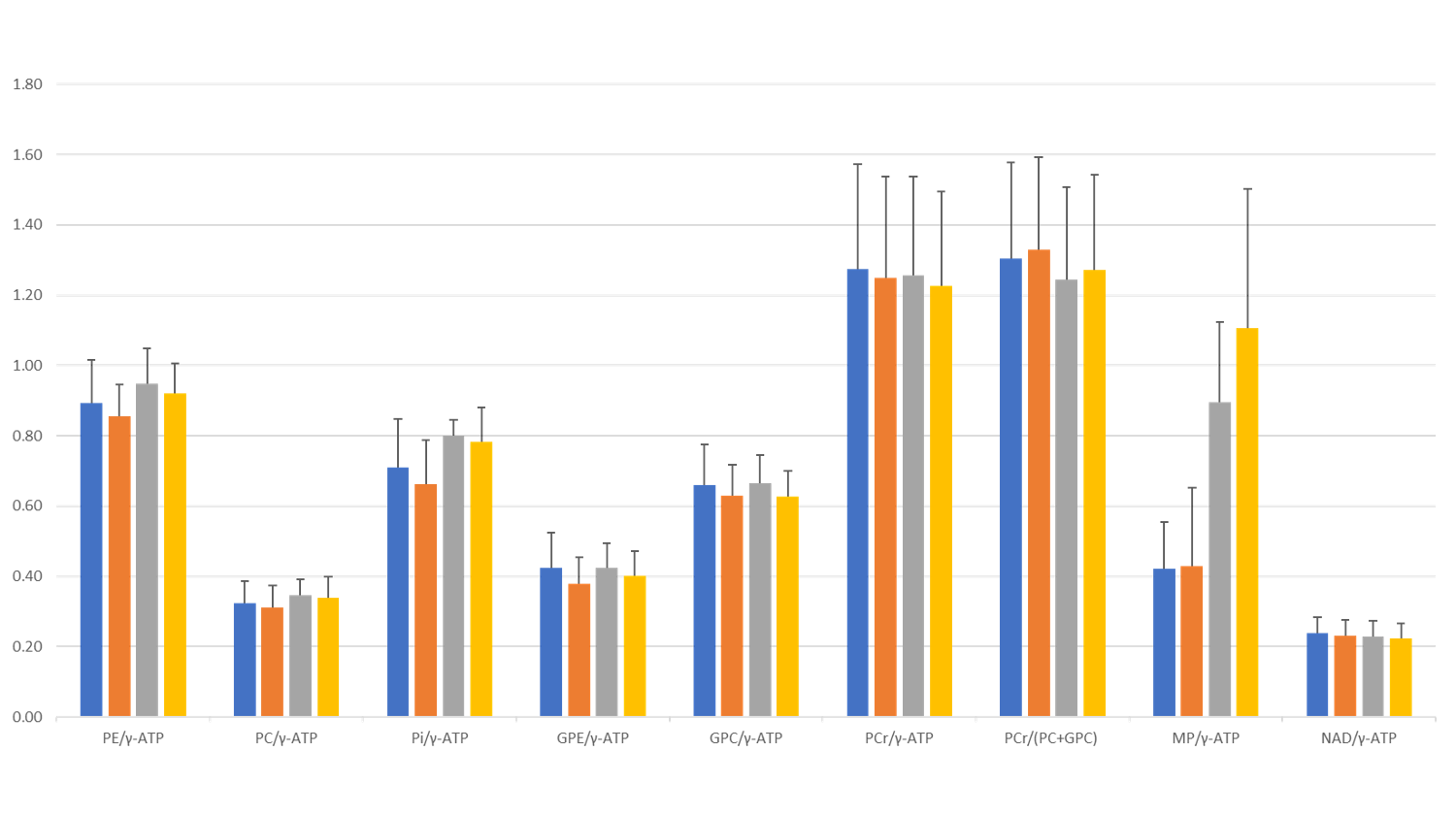

Supplement: S8 Fig — The MP signals were fitted using the four spectral models: Blue: Gaussian with a fixed linewidth of 100 Hz; orange: Gaussian with a freely adjustable linewidth; grey: Lorentzian with a fixed linewidth of 200 Hz; yellow: Lorentzian with a freely adjustable linewidth. (TIF) [file pone.0248632.s008.tif]
